# Effect of Restorative Material on Mechanical Response of Provisional Endocrowns: A 3D—FEA Study

**DOI:** 10.3390/ma14030649

**Published:** 2021-01-31

**Authors:** João Paulo Mendes Tribst, Alexandre Luiz Souto Borges, Laís Regiane Silva-Concílio, Marco Antonio Bottino, Mutlu Özcan

**Affiliations:** 1 School of Dentistry, University of Taubaté, Taubaté, São Paulo 12020-340, Brazil; joao.tribst@unesp.br (J.P.M.T.); regiane1@yahoo.com (L.R.S.-C.); 2 Institute of Science and Technology, São Paulo State University (Unesp), São José dos Campos, São Paulo 12220-690, Brazil; alexanborges@gmail.com (A.L.S.B.); mmbottino@uol.com.br (M.A.B.); 3 Division of Dental Biomaterials, Center for Dental and Oral Medicine, Clinic for Reconstructive Dentistry, University of Zurich, 8032 Zurich, Switzerland

**Keywords:** dental restoration failure, endodontically treated teeth, finite element analysis, dental materials

## Abstract

The goal of this study was to evaluate the stress distribution in an endocrown restoration according to different provisional restorative materials. An endodontically treated maxillary molar model was selected for conducting the finite element analysis (FEA), with a determined amount of dental remnant of 1.5 mm. The model was imported to the analysis software (ANSYS 19.2, ANSYS Inc., Houston, TX, USA) in STEP format. All contacts were considered perfectly bonded. The mechanical properties of each structure were considered isotropic, linear, elastic, and homogeneous. Three different provisional restorative materials were simulated (acrylic resin, bis-acrylic resin, and resin composite). An axial load (300 N) was applied at the occlusal surface in the center of the restoration. Results were determined by colorimetric stress maps of maximum principal stress, maximum shear stress, and total deformation. The different materials influenced the stress distribution for all structures; the higher the material’s elastic modulus, the lower the stress magnitude on the cement layer. In the present study, all provisional restorative materials showed similar stress patterns in the endocrown and on the cement layer however, with different magnitude. Based on this study limitation, the use of resin composite to manufacture provisional endocrowns is suggested as a promising material to reduce the stresses in the cement layer and in the dental tissue surfaces.

## 1. Introduction

The use of provisional restorations during dental treatment is an integral part of restorative procedures with indirect restorations, since it has important functions within the timeframe between preparation of a tooth and until fitting the final restoration [1]. In this sense, the adequate maintenance of the provisional restorations during all the dental treatment is extremely useful to promote an adequate oral health and can directly affect the ceramic restoration success [1]. To perform a function properly, the provisional restorations must meet esthetic, biologic, and also mechanical aspects, such as resistance to dislodging forces and chewing loads [2,3]. Therefore, clinical complications, such as catastrophic failures, should be avoided to not compromise the treatment longevity [3]. The restoration’s mechanical failure is usually justified in literature by the cyclic loading and chewing, subjecting the restorative material to various forces in oral conditions, including compressive force at the load application and tensile and shear stresses at the restoration’s intaglio surface [3,4]. The provisional restoration failure can cause economic loss and discomfort to both clinicians and patients, demanding repair procedures and sometimes a new provisional crown manufacturing [3].

It is well known that the provisional restoration’s resistance will be proportional with its anatomy and restorative material mechanical properties [4]. Basically, the provisional restoration can be manufactured using resin-based materials currently available—methacrylate resins, resin composite-based materials, and bis-acrylic resins [5]. Regardless of the chosen material, a precise fitting to the abutment tooth, proper flow, wetting, and plastic properties of the material are required [3,4,5]. However, the restoration volume can also affect how the restorative material will dissipate the chewing loads and the material mechanical response during stress situations.

One of the largest restoration volumes that can be used to restore a tooth is the endocrown design [6,7,8,9,10,11,12]. This restoration modality has emerged as a more conservative, quick, and easy-to-assemble approach, in which the build-up and crown integrate into a single structure and can be manufactured by computer-aided design using the pulp chamber as part of their mechanical retention [6]. Since the adhesive surface between abutment teeth and endocrowns is extended using micro-mechanical retention, the transmission of forces to dental abutments can be improved in comparison with a full crown [7]. However, this approach requires the presence of healthy cervical enamel and an adequate endodontic treatment in the abutment tooth [10].

Several studies have been carried out investigating the restorative material used to perform the endocrown treatment, reporting the use of ceramics, nanocomposites, and hybrid materials [6,7,8,9,10,11,12,13,14]. In addition, clinical cases reported the use of acrylic resin [15], resin composite [16], and bis-acrylic resin [17] as restorative material for the manufacturing of interim endocrowns. Nevertheless, the reason to use each material has not been deeply investigated and a comparison between them could be interesting for further studies and to elucidate the effect on the biomechanical behavior. Besides this, and since the benefits of having an adequate (esthetic, biologic, and also mechanical) provisional restoration are well known, the information regarding how the provisional materials affect the endocrown mechanical response is still scarce in the literature.

In order to understand the biomechanical behavior of endocrown restorations and the influence of restorative materials, a numerical simulation can be performed using the finite element method (FEM). This method has been extensively reported in literature, with previous studies that have evaluated this treatment modality [5,7,8,9,10,14,18,19]. As advantages, the FEM is able to identify problems by assessing the state of stress and deformation of materials and adhesive interfaces, evaluating the stress distribution generated by masticatory loads with model standardization, and offering an acceptable approximate solution [20,21,22]. Usually, failure origin consists of regions of high stress concentration, previously evidenced by the finite element analysis results [7].

Therefore, the objective of the present study was to evaluate the influence of restorative material (at three levels) on the biomechanical behavior of endocrown restorations. The null hypothesis was that there would be no difference between the restorative provisional materials for the endocrown biomechanical behavior.

## 2. Materials and Methods

A previous reported tridimensional first maxillary molar model was selected [7]. The use of a previous numerical model that has been compared with in vitro setup is important to guarantee the model validation in terms of failures. The file in standard for the exchange of product data (STEP) was exported to the modeling software (Rhinoceros version 4.0 SR8, McNeel North America, Seattle, WA, USA). The model was composed of the following geometries: cement layer (100 µm thickness) [6], dental preparation, and fixation cylinder [9]. In the present study, the purpose was to simulate the condition of a temporary endocrown prior to the final indirect restoration. The restoration intaglio surface was modified to allow all the contacting faces of the restoration and the cement layer to have similar number and shape, reducing the interference during the analysis [6]. The root model had a pulp chamber of 5 mm depth and a 16° wall inclination angle [7]. The finish line presented 1 mm of thickness. The endcrown presented a minimum thickness of 7 mm at the center of the occlusal surface [7] and 1.5 mm of sound enamel was considered [8].

**Figure 1:**
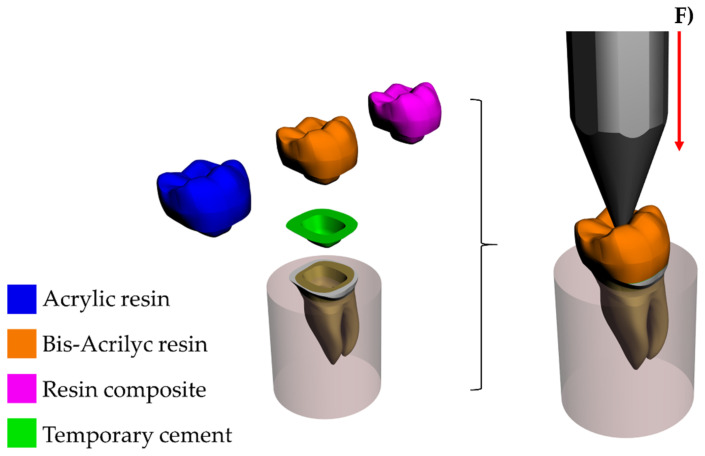
Three-dimensional model created in the modelling software with different provisional endocrown restorations.

**Figure 2:**
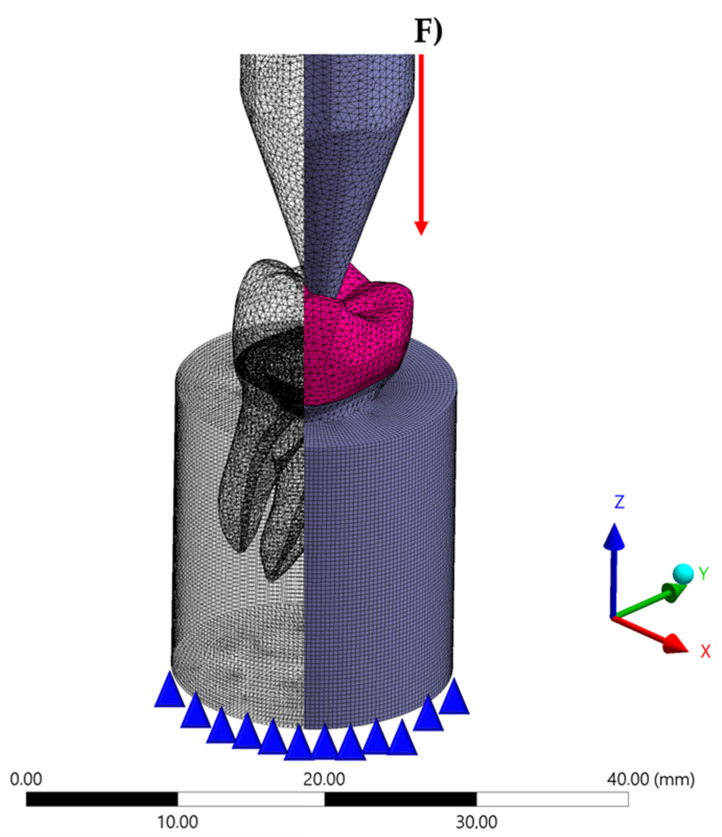
Finite element model after meshing process and boundary conditions applied in the simulation.

All geometries were verified as a volumetric solid without inconsistent normal or duplicate faces. Figure 1 shows the schematic illustration of the modeling. All models were exported to the analysis software (ANSYS 19.2, ANSYS Inc., Houston, TX, USA) and a convergence test of 10% mesh control determined the number of 98.653 nodes and 96.484 tetrahedral elements for the model (Figure 2). The aspect ratio of the mesh elements presented an average value of 1.8 ± 0.7 [7]. A conforming mesh across such interfaces ensures inter-element continuity in the finite element shape functions, resulting in a smooth and accurate interpolation of the numerical solution. Therefore, the mesh matching procedure was performed to remove the nonconforming interfaces. These elements allowed the stress transmission from the restoration to the cement, and, from the cement to the tooth with a reduced distortion caused by the mesh in the involved structures. Three different temporary restorative materials were chosen based in previous clinical cases that performed provisional endocrown restoration (acrylic resin, bis-acrylic resin, and resin composite). The mechanical properties of each material/structure (Table 1) were inserted into the analysis software and each material was considered isotropic and homogeneous.

As the analysis was performed considering a no-failure condition, all materials were assumed to behave as elastic materials. Bonded contacts were considered between the structures. In the boundary condition, the fixation occurred at the base of the polyurethane cylinder with fixed zero nodal displacements. Ensuring only the movement constraint on the Z-axis, the deformation generated in all directions could be computed. The occlusal loading (300 N) was applied to simulate a compressive load in the center of the cusps. For the restoration and the tooth adhesive area, the failure criteria was the maximum principal stress (in MPa). While for the cement layer, the tensile stress and shear stress were recorded. A qualitative stress map was generated from the postprocessing software, using color scales in these structures, and the highest peak in each structure was evaluated [6]. Differences with more than 10% were assumed as relevant between the models.

## 3. Results

**Figure 3:**
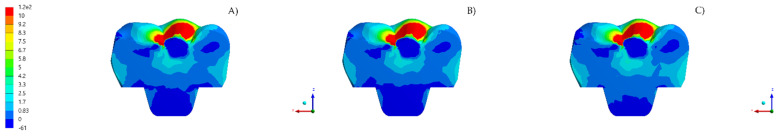
Tensile stress distribution in the sectioned endocrown according to the restorative material: (**A**) acrylic resin, (**B**) bis-acrylic resin, and (**C**) resin composite.

**Figure 4:**
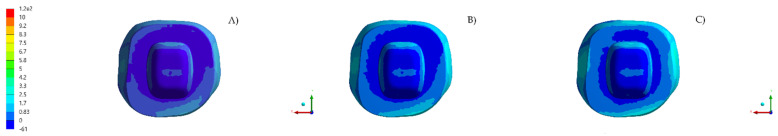
Tensile stress distribution in the endocrown’s intaglio surface according to the restorative material: (**A**) acrylic resin, (**B**) bis-acrylic resin, and (**C**) composite resin.

**Figure 5:**
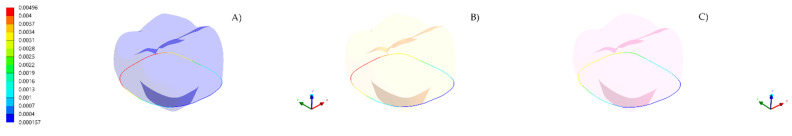
Displacement in the endocrown’s margin surface according to the restorative material: (**A**) acrylic resin, (**B**) bis-acrylic resin, and (**C**) composite resin.

**Figure 6:**
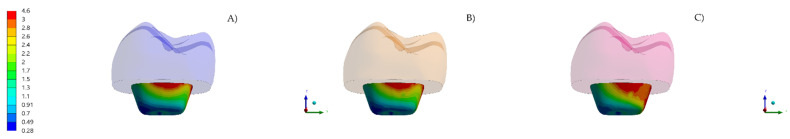
Maximum shear stress in the endocrown’s pulp-chamber extension according to the restorative material: (**A**) acrylic resin, (**B**) bis-acrylic resin, and (**C**) composite resin.

**Figure 7:**
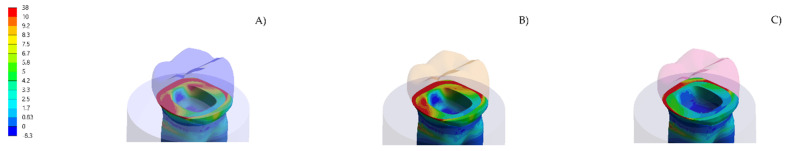
Tensile stress in the dental remnant according to the restorative material: (**A**) acrylic resin, (**B**) bis-acrylic resin, and (**C**) composite resin.

**Figure 8:**
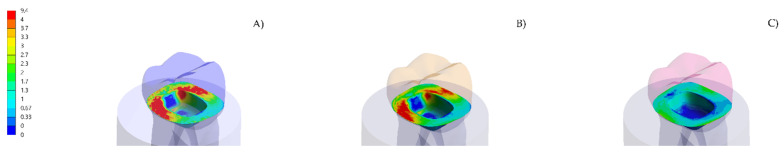
Tensile stress in the cement layer according to the restorative material: (**A**) acrylic resin, (**B**) bis-acrylic resin, and (**C**) resin composite.

**Figure 9:**
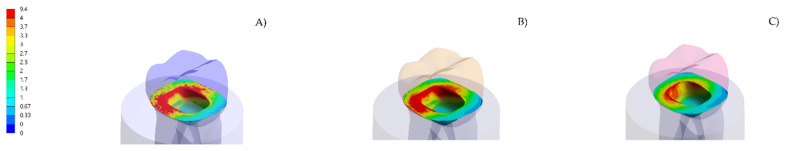
Maximum shear stress in the cement layer according to the restorative material: (**A**) acrylic resin, (**B**) bis-acrylic resin, and (**C**) resin composite.

After the processing, maximum principal stress (MPa) results were obtained for the endocrown, cement layer, and dental remnant structures. Maximum shear stress (MPa) results were obtained for the endocrown pulp chamber extension and the cement layer. Displacement (mm) was obtained for the endocrown margin. Data were summarized through colorimetric graphs (Figure 3, Figure 4, Figure 5, Figure 6, Figure 7, Figure 8 and Figure 9). For the endocrown restoration (Figure 3 and Figure 4), a similar stress distribution pattern was observed regardless the restorative material; however, the more rigid the temporary material was, higher stress concentration was observed in the endocrown (resin composite > bis-acrylic resin > acrylic resin). In addition, for the restoration marginal displacement (Figure 5), the endocrown in resin composite (0.0034 mm) was the less susceptible to deformation, suggesting the lowest chance to fail in this region, when compared to the endocrowns made in bis-acrylic (0.0041 mm) or acrylic resin (0.0042 mm). Observing the endocrown pulp-chamber extension (Figure 6), when the endocrown in resin composite was evaluated, the shear stress resin composite was higher than the endocrowns in bis-acrylic resin or acrylic resin, demanding higher bond strength in this region.

On the tooth surfaces (Figure 7), less stress concentration was observed with more rigid restorative materials. Basically, the material that concentrated more stress in the endocrown restoration showed less stress in the tooth adhesive surface (acrylic resin > bis-acrylic resin > resin composite). Regardless the restorative material, the enamel tissue concentrated more stress than the dentin. A similar behavior was observed for the cement layer, for both tensile and shear stresses (Figure 8 and Figure 9, respectively). In this sense, the resin composite endocrown seems to be less prone to adhesive failures in comparison with the other two materials.

A summary of means, standard deviations, minimum, and maximum values of the stress peaks is presented in Table 2.

## 4. Discussion

The null hypothesis was that there would be no difference between the restorative provisional materials for the endocrown biomechanical behavior. The results showed that restorative material influenced the biomechanical behavior of the provisional restorations. Thus, the hypothesis of this study was rejected. It was noticed that the material with the highest elastic modulus allowed less stresses to reach the cement layer, as a consequence of the restoration behavior [6]. In addition, previous investigation also indicated that the less rigid material could be an interesting alternative to perform this treatment modality [8]. This study corroborated both, showing that similar mechanical behavior was observed for the provisional restorative materials evaluated in the present analysis.

Besides the use of provisional endocrown being indicated for a short-term period, the restoration performance must be evaluated in order to guarantee all the provisional restoration clinical benefits and because any mechanical problem in the provisional restoration may cause discomfort for the patient, as well as financial and economic loss [20]. In this case, results that could suggest higher risk for marginal infiltration or restoration debonding would be valuable [9], since the occurrence of these failures during the usage of a temporary crown could be significant to negatively affect the treatment.

According to a systematic review [23], in which the success of the endocrown restoration was the primary outcome, the authors calculated a success rate for molars from 72.73% to 99.57% [23]. Moreover, the predominant mode of failure was an adhesive breakdown or the restoration debonding [23]. Despite the fact that this review has been performed using definitive restorative materials and long-term clinical trials, it showed the importance to evaluate the adhesive interface in this restorative modality. Observing the present results, the use of resin composite could offer to the patient and to the clinician a more reliable option in terms of marginal misfit and adhesive failure risk [24]. According to the literature [10], comparing different postendodontic treatments, the endocrown is able to promote higher tensile stress at the cement layer and in dentine, which increases the risk of failure by debonding [10]. The present study has complemented these findings, indicating that the provisional material can affect the endocrown mechanical response and should be selected in order to reduce the adhesive failure risk. The adhesive failure risk is proportional to the magnitude of stress concentration in the cement layer, tooth adhesive area, and restorative material. In addition, the internal adaptation can also be affected by the restorative material [11], affecting the cement layer thickness and, consequently, the polymerization shrinkage effect and voids content in it. In this investigation, all materials were simulated with an ideal fit and contact between the structures, which cannot occur clinically. Further studies should evaluate the influence of provisional endocrown material and manufacturing technique in the internal and marginal fit.

Endocrown margin infiltration has been previously evaluated in a 10-year retrospective evaluation of ceramic and composite endocrowns, the authors reported two cases of debonding and two cases of caries recurrence from the 99 evaluated cases [25]. Observing Figure 5, it is possible to observe that the higher the elastic modulus, the lower the deformation results. According to the literature, deformation between tooth and restorative material could lead to increased hydrodynamic flow and faster secondary caries lesion formation [26]. In addition, acrylic resin crowns can present a higher marginal discrepancy in comparison with resin composite provisional crowns due to a higher polymerization shrinkage effect [27]. Therefore, for the provisional step in the endocrown treatment, the use of resin composite should be suggested to reduce the endocrown marginal misfit during compressive load.

A previous reported model was used in this study and the remaining tooth presented 1.5 mm of sound enamel to provide adequate bond strength, attempting to preserve the maximum of the tooth structure [6,8]. It is also important to ensure that the prepared tooth must be able to be isolated so that optimal bonding protocols can be implemented under a rubber dam, especially because the debonding risk has been shown to be greater than the fracture risk [28,29,30].

In addition to the residual stress on the adhesive interface, the chewing load can also affect the mechanical response of the endocrown restoration [8]. In the present study, an axial load was applied due to the high prevalence of axial load in the molar region. However, the oblique incidence of chewing loads and cusp heights [9] also can affect the endocrown mechanical behavior and different occlusal anatomy can promote different results. However, for the molar region, the high incidence of axial loads in the posterior region makes the endocrown treatment a reliable modality considering the restoration fracture [12,14]. This study was also in agreement with this reported behavior, since the stress concentrations in the restoration cross-section and intaglio surface are not very different between the groups (Figure 3 and Figure 4). This similar stress distributions suggest that all of them will present a similar failure pattern during compressive tests. During the preparation design, increasing the restoration occlusal thickness can promote a significant improvement in fracture strength of ceramic endocrowns [29,30,31]. However, for provisional restorations, this information is not available in literature yet. Another aspect of the preparation design is the use of a flat pulp-chamber extension with radicular extension, which can increase the fracture strength; however, it promotes more unfavorable fracture patterns [29,32]. To ensure an easy preparation, filling of the pulp chamber with flowable resin composite should be applied before the provisional endocrown restoration manufacturing, similar to the present model design.

Another aspect of the endocrown design to think about is the walls of the central retainer in the endocrown’s pulp-chamber extension promoting an adequate load dissipation [32]. Therefore, the smoothening of the sharp edge at the base of the pulp chamber in the preparation for endocrowns is suggested to reduce the stress concentration in the cervical dentin beneath the endocrown, as well as on the cement layer [33]. This study followed this approach in the endocrown modelling and showed that the magnitude of shear stress concentration can be proportional to the restorative material elastic modulus. The resin composite will demand more bond strength in this region when compared with the other materials; however; it also has the highest bond strength between all of them.

One of the advantages of the endocrown treatment is that molars restored with endocrowns are less prone to root fracture than those with posts [34]. This can be explained because the stress concentration occurs in the occlusal plane from the prepared abutment tooth [6,7,8,9,10]. Another aspect is that the stress distributions in the root were similar regardless the ceramic restorative material and different occlusal clearance when indirect restorations were used [34]. In addition, the present study suggests that the same behavior could be observed in the abutment tooth when a provisional endocrown was lutted on it; however, the stress pattern and magnitude were proportional to its elastic modulus. It is expected restorations that mimic the natural tooth structures will survive longer in oral conditions [34], and therefore, a biomimetic restoration can provide an adequate stress distribution [35]. The resin composite group can be suggested for a similar approach in provisional endocrown restorations (using layering techniques and resin composites with different filling contents) allowing the dental technician and dentist to obtain an optimal provisional endocrown. However, it should be evaluated in further studies, modifying the endocrown layers’ mechanical behavior.

According to the literature [36], material characteristics may influence the endocrown’s margin stability, and for long-term restorations, computer-aided design/computer-aided manufacturing (CAD/CAM) resin-based composite materials can present a better aspect of marginal fit than ceramic materials [35]. Therefore, this benefit can also achieved for the provisional step of the treatment with the resin composite group. Still comparing different endocrown restorative materials, a previous study evaluated the stress distributions of endocrown molar models fabricated with five materials by finite element method and load-to-fracture test. Although the authors focused on studying definitive CAD/CAM restorations, they concluded that the endocrown fabricated with resin composite exhibited the best monobloc stress distribution and met the mechanical requirements for large occlusal areas [37]. In this sense, the resin composite restorative materials seem to be an optimal suggestion, even for long-term restorations, corroborating the present study results. In addition, resin composites are reported to be superior in their ability to maintain constant deformation without excessive dissipation of stress when compared to acrylic resins [38]. However, for resin composite endocrowns manufactured with a layering technique, this information is not available in the literature and, therefore, the results should be carefully extrapolated. In agreement with that, in a previous report [20] that evaluated provisional restorations, the authors found that the acrylic resin showed a more accurate and precise marginal adaptation in comparison with bis-acrylic, however they justified it based in the CAD/CAM manufacturing method. Therefore, different manufacturing techniques can modify the mechanical response of endocrowns manufactured with the same restorative material and should be considered during the treatment plan. Due to the fact that this is a numerical simulation, the 3D model does not present the defect population in its structure and a homogeneous material was considered, therefore the digital workflow can produce endocrowns with higher similarity with the present study in comparison with the analog method.

There are different clinical studies that have reported the use of acrylic resin [15], resin composite [16], and bis-acrlyc resin [19] as restorative material for the manufacturing of provisional endocrowns. Until now, there has been no information regarding the difference between these reported materials. Therefore, the present study suggests that resin composite should be mechanically more adequate to be used for this indication. A clinical report asserted that the temporary endocrown restoration could be performed with self-polymerizing acrylic to promote an adequately adapted gingival margin and to ensure healthy gingival tissue [39]; however, the authors did not evaluate these properties in the study and the present study only considered the mechanical response. Therefore, further clinical trials evaluating the gingival tissue between different interim restorative materials for endocrown treatment are suggested to elucidate this biological aspect.

A previous study aimed to evaluate and compare the marginal accuracy of interim restorations made from acrylic resin and bis-acryl resin materials [40]. The authors found that the bis-acryl group showed a better marginal accuracy than the Polymethyl methacrylate (PMMA) group. The authors justified these results based in the higher volumetric polymerization shrinkage for PMMA (6%) when compared with 1% to 2% for composite materials [40]. Therefore, the present study complement this information, showing that even after the polymerization and luting procedure, the marginal deformation will be affected by the restorative material stiffness.

According to the literature [6,8,18,19], the greater the stiffness of the endocrown restorative material, the lower the stresses recorded in the tooth structures as well as the stresses at the interface between these restorations and the dental tissue. This same pattern has been observed in the present results, corroborating the resin composite material indication during the provisional manufacturing. As the resin composite endocrown should be manufactured by CAD/CAM or by layering technique, more time and effort will be applied in its manufacturing in comparison with the other two provisional materials (acrylic and bis-acrylic). However, according to the literature [41], an interim restoration, even for a brief period, will assist the clinician in evaluating the treatment plan and improving the prognostic accuracy of prosthetic treatment. The initial investment will be more than repaid in time saved for later procedures, adjustments, and remakes.

The literature reports that endocrowns appear to be a promising conservative restorative modality with acceptable long-term survival for endodontically treated posterior teeth in selected patients [38]. The present study corroborates with that, showing low stress magnitude regardless the restorative material used in the provisional step. However, because this is an in silico simulation, some limitations derived from the applied methodology were present. Initially, the restoration would be subject to variations in temperature and pH in an oral cavity. In addition, the simulated materials were considered isotropic and do not present defect populations. Vertical misfits of the prostheses were not simulated, as well as oblique load application, sliding contacts, and operator errors. However, further studies should be carried out to complement the present findings, assisting in elucidating the provisional endocrown’s biological behavior, fracture load, fatigue survival, and marginal infiltration, as well the marginal and internal adaptation of each material, followed by clinical studies.

## 5. Conclusions

In the present study, all provisional restorative materials showed similar stress patterns in the endocrown. The higher the provisional material elastic modulus, the lower was the stress concentration in the cement layer. Based on this study limitation, the use of resin composite to manufacture provisional endocrowns is suggested as a promising material to reduce the stresses in the cement layer and in the dental tissue adhesive surfaces.

## Figures and Tables

**Table 1:** Mechanical properties of the materials/structures used in the current study.

Material/Structure	Composition	Elastic Modulus (GPa) *	Poisson Ratio	Reference
Enamel	-	80	0.30	[22]
Dentin	-	18	0.23	[22]
Fixation cylinder	Polyurethane resin	3.6	0.30	[5]
Temporary cement	Zinc oxide-based cement	1.35	0.30	[23]
Acrylic resin	Polymethyl methacrylate, diethyl phthalate, benzoyl peroxide, titanium dioxide.	2.2	0.30	[5]
Bis-acrylic resin	UDMA, bis-GMA, benzoyl peroxide, Amine and fillers.	2.9	0.30	[5]
Resin composite	UDMA, bis-GMA, bis-EMA, TEGDMA, Silica and fillers.	8.0	0.25	[22]

* Values obtained from the literature.

**Table 2:** Stress peaks (MPa) obtained in each structure after the analysis processing.^1^

	Acrylic Resin	Bis-Acrylic Resin	Resin Composite
Tensile stress in the endocrown	9.6	9.7	10.1
Shear stress in the endocrown	4.2	4.3	4.7
Tensile stress in the cement layer	8.9	8.8	3.4
Shear stress in the cement layer	9.2	9.0	7.5
Tensile stress in the enamel tissue	16.5	16.1	13.4
Tensile stress in the dentin tissue	15.9	15.2	9.3

^1^ Stress peaks automatic identified by the software.

## Data Availability

The data presented in this study are available on request from the corresponding author.
